# Complete Genome Sequence of the Marine-Derived Bacterium *Streptomyces* sp. Strain GMY02

**DOI:** 10.1128/MRA.00681-21

**Published:** 2021-10-07

**Authors:** Jaka Widada, Ema Damayanti, Mustofa

**Affiliations:** a Department of Agricultural Microbiology, Faculty of Agriculture, Universitas Gadjah Mada, Yogyakarta, Indonesia; b Research Division for Natural Product Technology, Indonesian Institute of Sciences, Yogyakarta, Indonesia; c Department of Pharmacology and Therapy, Faculty of Medicine, Public Health and Nursing, Universitas Gadjah Mada, Yogyakarta, Indonesia; Montana State University

## Abstract

We report the complete genome sequence of *Streptomyces* sp. strain GMY02, isolated from Indonesian marine sediment. This bacterium has a circular 8,512,626-nucleotide chromosome. Genome mining analysis of the whole-genome sequence revealed that GMY02 has 28 biosynthetic gene clusters, dominated by genes encoding nonribosomal peptide synthetase and polyketide synthase.

## ANNOUNCEMENT

Marine actinobacteria are biotechnologically valuable organisms sourced from the ocean (1). Various marine actinobacteria, especially those from the genus *Streptomyces*, have been isolated from marine sediments and sponge species (2). In the past 10 years, the discovery of new compounds from marine *Streptomyces* bacteria have led to the further development of novel antibacterial (3), antiproliferative (4), antimicrobial (5), antibiofilm (6), anticancer-antitumor (7), and antiplasmodial (8) and anticomplement compounds (9). *Streptomyces* strain GMY02 is a potential bacterium which was isolated from a marine sediment sample from Krakal Beach (8°8′44″S, 110°35′59″E), Yogyakarta, Indonesia, using the method described in a previous study (10). GMY02 was isolated using solid starch nitrate medium, which was prepared using seawater and contained 75 μg/ml cycloheximide. In this study, we present the sequence, assembly, and annotation of the whole-genome sequence (WGS) of the bacterium GMY02.

The genome of GMY02 was obtained from cells grown from a lyophilized culture in the laboratory, which were then grown in tryptic soy broth (TSB) medium at an incubation temperature of 30°C for 48 h and recultured on International *Streptomyces* Project-2 (ISP-2) agar medium at 30°C for 5 days. High-molecular-weight (HMW) genomic DNA of the bacterium was isolated using the Nanobind CBB big DNA kit (Circulomics). The DNA concentration was determined using both NanoDrop spectrophotometers and a Qubit fluorometer. The library preparation was conducted using kits from Oxford Nanopore Technology. Nanopore WGS data were obtained using GridION sequencing with MinKNOW version 20.06.9 software. Base-calling was performed using Guppy version 4.0.11 with high accuracy mode (11). All FASTQ files were filtered using Filtlong software (https://github.com/rrwick/Filtlong), and the quality was visualized using NanoPlot (12). *De novo* assembly was conducted using Flye version 2.8.1 software (13) (average read length, 4,046 bp; total number of reads, 522,890). Medaka software (ONT Research; https://github.com/nanoporetech/medaka) was used for polishing the assembled sequence. The assembled contig was aligned to the reference genomes of type strains using Mauve version 2.4.0 (14). The assembled genome sequences and their annotation were assessed using both BUSCO (15) and CheckM (16) software. The genome completeness was determined to be 99.05%, with 0.51% contamination, and the average sequence coverage was ∼138×. Default parameters were used for all software unless otherwise noted.

Full-genome sequencing of strain GMY02 led to an assembly of 1 contig for a total genome size of 8,512,626 Mbp and a GC content of 70.4%. Annotation was conducted using the NCBI Prokaryotic Genome Annotation Pipeline (PGAP) version 5.2 (best-placed reference protein set; GeneMarkS-2+; https://www.ncbi.nlm.nih.gov/genome/annotation_prok/) and identified a total of 7,194 genes, including 7,098 coding DNA sequences (CDSs), 96 RNA genes, 69 tRNAs, and 9 noncoding RNAs. The average nucleotide identity based on BLAST+ (ANIb) using JSpecies Web Server (JSpeciesWS) (17) showed that *Streptomyces* sp. strain GMY02 and Streptomyces odonnellii strain NRRL B-24891 (GenBank accession number NZ_LATD00000000.1) had ANI values of 94.84%. Genome mining analysis using antiSMASH version 6.0 (18) revealed that GMY01 has 28 regions of biosynthetic gene clusters (BGCs).

### Data availability.

This whole-genome shotgun project has been deposited at DDBJ/ENA/GenBank under the accession number CP077658, BioProject accession number PRJNA737600, and BioSample accession number SAMN19700779. The raw sequence reads are available under SRA accession number SRP326726. The version described in this paper is CP077658.1.
